# NewSTEPs: The Establishment of a National Newborn Screening Technical Assistance Resource Center

**DOI:** 10.3390/ijns4010001

**Published:** 2017-12-22

**Authors:** Jelili Ojodu, Sikha Singh, Yvonne Kellar-Guenther, Careema Yusuf, Elizabeth Jones, Thalia Wood, Mei Baker, Marci K. Sontag

**Affiliations:** 1 Association of Public Health Laboratories, 8515 Georgia Avenue, Suite 700, Silver Spring, MD 20910, USA; 2 Colorado School of Public Health, University of Colorado Denver, Anschutz Medical Campus, Aurora, CO 80045, USA; 3 Wisconsin State Laboratory of Hygiene and Department of Pediatrics, University of Wisconsin School of Medicine and Public Health, Madison, WI 53706, USA

**Keywords:** newborn screening, data repository, continuous quality improvement, evaluation, technical assistance

## Abstract

As newborn screening (NBS) programs in the US implement expanded screening panels, utilize emerging technologies and identify areas for improvement, the need to establish and maintain a community engagement based national technical assistance center becomes apparent. The Newborn Screening Technical assistance and Evaluation Program (NewSTEPs)—a program of the Association of Public Health Laboratories (APHL) in partnership with the Colorado School of Public Health (ColoradoSPH), offers expertise in newborn screening program development, member connection, data analysis, and program evaluation. NewSTEPs provides a secure online data repository designed to collect comprehensive data on newborn screening programs in three strata: state profiles (description of each state program including program hours, fees, and disorders screened), quality indicators (metrics of program performance encompassing screening accuracy and timeliness) and NBS public health surveillance case definitions. NewSTEPs was created in 2012 under a cooperative agreement with the United States Department of Health and Human Services (HHS), Health Resources and Services Administration (HRSA), Maternal and Child Health Bureau (MCHB). Successful activities of NewSTEPs have resulted in the establishment of a technical assistance resource center and the organization of a network of newborn screening experts. In addition, NewSTEPs coordinates efforts with other federally funded programs in order to maximize resources and to ensure a unified approach to data collection and information sharing.

## 1. Introduction

Public health laboratories in the United States screen for heritable conditions in approximately four and a half million newborns every year [1]. While the Health and Human Services (HHS) Secretary’s Advisory Committee on Heritable Disorders in Newborns and Children (ACHDNC) recommends disorders to be included on the Recommended Uniform Screening Panel (RUSP), each state determines the specific disorders for which it screens [2]. As of December 2017 all states universally screened for at least 28 of the 34 disorders on the RUSP [3]. The differences amongst programs can be understood by examining a variety of categories (regional laboratories, one screen versus two screen states, legislatively driven programs) with patterns emerging that demonstrate the dynamic state of NBS and related policies in the United States. Historically a lack of standardization among states coupled with disparate data definitions has limited the ability to evaluate quality across the national newborn screening system [4]. Newborn screening programs have utilized locally developed data definitions for defining a true positive case following NBS and for measuring the success of their programs through quality metrics. Establishing and maintaining a centralized repository of standardized data elements collected consistently across NBS programs and producing routine reports allows programs to conduct self-peer comparison and identify the needed improvement areas. NewSTEPs, the Newborn Screening Technical assistance and Evaluation Program, offers this uniformity with community-driven data definitions.

The Health Resources and Services Administration (HRSA) Maternal and Child Health Bureau (MCHB) promotes improving the health of infants, children, mothers, and families. HRSA funded the development of the Council of Regional Networks for Genetic Services (CORN) in 1985 to provide a forum for exchange among groups concerned with public health aspects of genetic services. Upon disbanding CORN in 1999, HRSA established the National Newborn Screening and Genetics Resource Center (NNSGRC), supported by a cooperative agreement between the Genetic Services Branch of HRSA’s MCHB and the University of Texas Health Science Center at the San Antonio Department of Pediatrics [5]. The NNSGRC was funded by HRSA until 2012 to enhance the quality of newborn screening and related genetic services in the United States.

Building on these previous activities and with an emphasis on establishing consistency in data collection, supporting implementation of quality improvement practices and program evaluation, the Newborn Screening Technical Assistance and Evaluation Program (NewSTEPs) was launched in 2012. NewSTEPs is funded through authorization under the Public Health Service (PHS) Act, § 1109 (42 U.S.C. 300b-8). NewSTEPs is a program of the Association of Public Health Laboratories (APHL), in partnership with the Colorado School of Public Health (ColoradoSPH). The Association of Public Health Laboratories is a member organization that represents Public Health Laboratories, equipping it as a logical technical assistance resource serving the needs of state newborn screening laboratories, follow-up programs and stakeholders. 

NewSTEPs’ data elements reflect the collection of Healthy People 2020 Maternal, Infant and Child Health Morbidity and Mortality Health Services Objectives [6] and its program activities align with the Newborn Screening Saves Lives Reauthorization Act [7] goals of supporting expansion of national NBS programs and ensuring quality laboratory and program practices.

This report describes the components of a comprehensive technical assistance and resource center for newborn screening and the regular evaluation activities designed to assess the success of the program. The model described can be adopted by other public health and clinical programs to support systematic efforts to improve outcomes by supporting standardized data collection and sharing of resources.

## 2. Methods

### 2.1. Standardizing Data Nationally

Since receipt of Cooperative Agreement #U22MC24078 from HRSA on 1 June 2012, NewSTEPs has led a national effort to improve the NBS system with a focus on standardization, data-driven quality improvement, evaluation, bidirectional communication, and information sharing. NewSTEPs engages in partnership strengthening with stakeholders throughout the newborn screening community, with NewSTEPs core activities offering data collection, information dissemination, quality monitoring tools, and quality improvement resources.

### 2.2. Establishing Governance

In 2012 the NewSTEPs steering committee was established, consisting of NBS experts who were invited to join based on leadership skills in the newborn screening community, with a balanced representation from different aspects of the NBS system, with expertise in a variety of fields (e.g., information technology, medical, follow-up, genetics, and laboratory testing), and with members serving a rotating three-year term. The steering committee met monthly via webinars and in-person at least once per the Cooperative Agreement Cycle. The steering committee, along with NewSTEPs staff and HRSA representatives, set priorities and led activities for subsequent growth. 

NewSTEPs convened workgroups, each with a distinct focus, to guide the activities of the program including website content, the data repository data elements and definitions, quality indicators, newborn screening public health standard surveillance case definitions, disorder specific activities, and technical assistance. The workgroups were comprised of balanced representation from newborn screening laboratory, follow-up and clinical disciplines. The purpose and duration of each workgroup is presented in Table 1.

### 2.3. Relationship Building and Community Engagement

The collaborative nature of NewSTEPs activities are built off of APHL’s existing relationships with partners and state public health laboratories and are driven by active interactions with NBS programs, pediatric subspecialists, and stakeholders from the MCHB of HRSA funded programs including the national newborn screening clearinghouse, Baby’s First Test, Regional Genetic Service Collaboratives, and their National Coordinating Center. NewSTEPs also works with the Newborn Screening Translational Research Network (NBSTRN), funded through the National Institutes of Health and partners with the Centers for Disease Control and Prevention (CDC). 

NewSTEPs program staff held targeted discussions with partners in 2012 and thereafter on an ongoing basis, including the American College of Medical Genetics and Genomics (ACMG), the Genetic Alliance, the National Library of Medicine (NLM), among others to share NewSTEPs’ goals (Table 2), establish mechanisms to address common priorities without duplicating efforts, and to consider the incorporation of subspecialty feedback into the data collection efforts. 

To assess the needs of NBS programs, NewSTEPs program staff issued an online data use survey (Table 3) in 2012 to identify gaps in and desired attributes of comprehensive NBS programs. Incorporation of stakeholder feedback, including modifications to existing quality indicators and scope of Newborn Screening Public Health Surveillance Case Definitions, into NewSTEPs activities has been a step toward establishing trust within the newborn screening community. 

### 2.4. Consensus Building

NewSTEPs pursued consensus building activities for information exchange as well as for the development of data elements to populate the NewSTEPs Data Repository. Timely information exchange related to program, partner and state activities occurred via meetings, through trainings, by information exchanged on a dedicated listserv, and via the interactive website, www.newsteps.org. These communication mechanisms were identified as trusted information resources in interviews with key stakeholders—six members of the NBS community representing laboratory, follow-up and clinical practice—conducted by NewSTEPs in 2013.

Consensus building and identification of necessary data elements culminated in the development of requirements that the NewSTEPs Data Repository should entail. The most significant demonstrations of need resulting from these stakeholder interactions were: (1) the need for a robust data repository; (2) the need for data to be collected in a standardized, comparable and quantifiable manner; (3) and provision to NBS programs of data sharing agreements addressing data privacy and security requirements. 

### 2.5. Data Use and Data Privacy Requirements

In order to address data use and data privacy requirements, NewSTEPs sought guidance on all data sharing activities from the Colorado Multiple Institutional Review Board (COMIRB); and the Department of Health and Human Services (HHS) Office of Human Research Protection (OHRP). Both entities deemed this project to be Non-Human Subject Research, with supporting letters posted on the NewSTEPs website. With NewSTEPs not participating in human subject research, the individual entities (states) entering data into the repository would also not be participating in human subject research, therefore there was no need to obtain consent at the infant level or to obtain Institutional Review Board (IRB) approval from individual states. This was an important hurdle to overcome in gathering data.

### 2.6. Data Use Survey

The newborn screening needs survey was completed by 34 individuals (newborn screening follow-up staff (*n* = 10), laboratory staff (*n* = 3), newborn screening program supervisors (*n* = 11), clinicians (*n* = 2), Genetics Regional Collaborative members (*n* = 6) and other newborn screening stakeholders (*n* = 2). Responses enabled NewSTEPs program staff to consider what elements would make the NewSTEPs Data Repository a useful resource for the community; collated responses are provided in Table 3. The survey elucidated a series of needs throughout the system. The gaps in newborn screening technical assistance and quality improvement ranged from a lack of support for implementing newborn screening for new conditions to a paucity of short-term follow-up networking and support.

## 3. Results

### 3.1. Data Repository

The NewSTEPs Data Repository was developed as a centralized and secure database designed for NBS programs to explore data to meet local program needs. NewSTEPs used consensus building to define and collect three levels of data for the data repository: state profiles, quality indicators, and case data. The NewSTEPs Data Repository was launched in May 2013, with additional releases occurring quarterly. The categories of data elements collected within the repository are listed in Table 4, encompassing the breadth of the NBS system. The NewSTEPs Data Repository is web-based, meeting stringent security standards, and can be accessed by authorized users, with delineated and multiple layers of access, from anywhere, allowing each NBS program to securely explore data to meet local program needs.

The NewSTEPs Data Repository has well-defined user roles. Each NBS program has a state administrator role, one that manages all users for their state. NBS programs that enter data into the NewSTEPs Data Repository can access their own data, including case-level data, and quality indicators. They can also view aggregate data from other participating NBS programs. Only NewSTEPs staff can access all state level data to develop aggregate state, regional and national reports. Public access has been limited to the state profiles on the NewSTEPs website because of the desire of states to maintain the privacy of their data. In order to submit data into the NewSTEPs Data Repository, NBS programs are required to sign a memorandum of understanding (MOU) with APHL. The decision was made to have an MOU with states so that the newborn screening program could demonstrate it had permission from their larger organization to share the data and to make clear how the data would be shared and utilized. The MOU describes data ownership, data reporting and data security and is available on the NewSTEPs website. To facilitate streamlined data entry and to provide a more standardized approach to data definitions, NewSTEPs has partnered with Laboratory Information Management Systems (LIMS) vendors and Health Information Technology (HIT) experts in state public health departments to develop data import templates. For many state programs, this step was necessary in gaining their partnership. As of December 2017, 44 states have signed MOUs to enter data into the NewSTEPs Data Repository. Additionally, all 50 states, Puerto Rico and Washington, DC have entered state profile data, 40 states have entered or provided quality indicator data, and 29 states have entered case definition data.

### 3.2. Newborn Screening Program Site Reviews

NewSTEPs conducts non-regulatory site review visits on an as-requested basis, using a site review tool and process adapted from other sources. Site review needs are assessed by responses provided to a pre-site review survey, which was developed by the NewSTEPs Evaluation Work Group and is completed by Laboratory and Follow-Up Directors of each requesting program. The customized site review visits are aimed at assessing various components of a NBS program including the laboratory system, birth facilities, and follow-up system for quality improvement purposes. The comprehensive site visit is conducted by a team of experts that review programs in a customizable manner, with a focus on assessing programmatic areas including state legislation and policy, ethics, funding models, organizational structure, and education.

Following a site review visit, the host NBS programs receive a written report from NewSTEPs, comprised of actionable recommendations and measurable outcomes. The report captures information on the NBS program, including general perceptions, an assessment of the functions of each component of the screening program (pre-analytical, analytical, and post-analytical), and a list of recommendations for future program changes and growth. Between 2012 and 2017, NewSTEPs has comprehensively evaluated seven programs and conducted a focused review in one program.

### 3.3. Quality Indicators

NewSTEPs adopted and refined a panel of eight quality indicators designed by a panel of expert stakeholders with the purpose of providing longitudinal comparisons within a NBS program and comparisons to aggregate data across NBS programs. The quality indicators were initially developed under the leadership of the HRSA Genetic Services Branch and were refined through a series of stakeholder meetings and webinars led by NewSTEPs. The quality indicators (Table 5) will be reevaluated every three years. As of December 2017, 32 states have entered state level quality indicator data, enabling comparisons within and across programs, in aggregate.

### 3.4. Public Health Standard Surveillance Case Definitions

NewSTEPs expanded on activities initiated by HRSA and the clinical subspecialist community by integrating existing public health surveillance case definitions in the data repository and developing worksheets and a toolkit for NBS programs to collect information from clinical specialists who confirm cases of infants identified through NBS. Implementation of the case definitions allows for uniform comparisons to be made across and within NBS programs for the purposes of estimating incidence and developing quality improvement initiatives. By December 2017, 29 states have entered over 7900 cases into the NewSTEPs data repository.

### 3.5. New Disorder Technical Assistance

NewSTEPs has utilized information exchange among NBS programs to provide technical assistance, particularly for the implementation of new disorders added to the RUSP. As disorders are added to the RUSP, the NBS programs that have begun implementation pave the way for the rest of the community. Since its inception, NewSTEPs has provided resources and technical assistance for critical congenital heart disease (CCHD), severe combined immunodeficiency (SCID) [Cooperative Agreement #UG5MC27837 from HRSA], Pompe disease, Mucopolysaccharidosis I and X-linked adrenoleukodystrophy [Cooperative Agreement #UG9MC30369 from HRSA]. Technical assistance offerings have been formalized by the creation of short-term follow-up, CCHD, SCID and new-disorder-specific focused workgroups (Table 1).

Resources that have been provided for these disorders include educational information, screening status reports, legislative updates, pertinent publications, suggested data elements, and best practices—all of which are housed on the NewSTEPs website. NewSTEPs also hosts disorder specific webinars to address the needs of stakeholders in the states. Additionally, NewSTEPs hosts disorder specific national meetings to facilitate information sharing and to offer guidance for NBS programs preparing to implement.

### 3.6. Data Reporting

State profile data are summarized, updated in real time and are available on the public-facing NewSTEPs website in the form of interactive maps, tables and reports. Examples of publicly available data are: the status of newborn screening for new conditions, validation and pilot study status, screening methodologies and targets, NBS fees, dried blood spot retention, courier system, LIMS system, and operating hours. 

Registered users from state newborn screening programs can utilize standardized reports to review more detailed information on newborn screening programs, allowing comparison of their own state’s data to the outcomes of other states. To ensure program confidentiality, state identities are blinded and the personnel from each state only know their own state’s identity. Most recently, NewSTEPs has incorporated Tableau Software to provide interactive infographics of the three strata of data collected (state profiles, quality indicators and case data). These infographics are available on the NewSTEPs website and maintain the program confidentiality as appropriate.

### 3.7. Sharing Resources

NewSTEPs offers resources in the following categories: Quality Practice Resources, Site Review Program, News and Education, Reports, State Profiles, NewSTEPs Data Repository and Data Infographics. These are shared on the NewSTEPs website (www.newsteps.org) which was launched in May 2013. The website is designed with a focus on content geared toward NBS program personnel and medical professionals. The NewSTEPs website houses technical assistance resources including toolkits, webinars, data infographics and best practice strategies for disorders added to the Recommend Uniform Screening Panel. Archived videos and transcripts from NewSTEPs national webinars are also are available to assist programs with continuous quality improvement and ongoing education.

### 3.8. Role as Conveners

NewSTEPs incorporated feedback from the Data Use Survey to initiate a Short-Term Follow-Up workgroup, a forum that did not previously exist on a national level. This is an example of the role of NewSTEPs in convening stakeholders on a routine basis, via workgroup, webinars and in-person meetings, to share ideas, discuss challenges and identify solutions to barriers. Table 6 depicts national in-person meetings convened by NewSTEPs, with the guidance of the NewSTEPs steering committee and the workgroups outlined in Table 1.

## 4. Discussion

Since its inception in 2012 NewSTEPs has established its presence and value within the newborn screening community, playing a key role in information dissemination via a well-utilized NewSTEPs listserv as well as through routine technical assistance webinars aimed toward various subsections of the broad newborn screening community comprised of laboratorians, follow-up program staff, clinicians, vendors and parents.

NewSTEPs has expanded on previous quality improvement efforts by providing data, technical assistance, and educational resources to NBS programs. NewSTEPs has approached the issue of NBS harmonization using technology and innovation, including a comprehensive data repository used for evaluating outcomes. NewSTEPs has also developed real-time data infographics that allow programs to visualize the impact of their quality improvement processes. Much of the success of NewSTEPs has been the result of community engagement and incorporating feedback into all its activities.

In conclusion, NewSTEPs is playing a critical role in ensuring that NBS programs can adequately evaluate, analyze, and benchmark the performance of their tests and the quality of their activities. To be effective and successful, NBS systems require partnerships that include families, health care providers, and local, regional, state, national and private organizations. The activities of NewSTEPs are designed to build partnerships with the ultimate goal of improving quality in the NBS system.

## 5. Conclusions

The structure that has been implemented by NewSTEPs can be adopted by other public health programs to support coordinated efforts for improving programs at the state and regional levels. Specifically, community engagement in developing standard data elements and identifying technical assistance needs has enabled NewSTEPs to engage with newborn screening programs on a national scale in support of continuous quality improvement.

## Figures and Tables

**Table 1:** Newborn Screening Technical assistance and Evaluation Program (NewSTEPs) Workgroups (2012–Present).

Workgroup	Dates of Activity	Purpose and Activities
Evaluation Tool	2012–2013	To assist with developing a pre-site evaluation tool and a formal, comprehensive site evaluation tool, building on previous documents [8].
Website Content	2012–2013	To develop an outline of the content types, primary audience, and features that the NewSTEPs website would exhibit.
Data Repository	2013–2014	To Identify necessary requirements and data elements to be captured within the NewSTEPs Data Repository.
Quality Indicators I	2012–2013	To refine newborn screening quality indicators developed by HRSA in 2011.
Quality Indicators II	2015–2016	To continue to refine and to improve the conceptual definitions of the QIs and to identify mechanisms to facilitate easier data entry for these QIs into the NewSTEPs data repository.
Case Definition Implementation	2015–Present	To develop strategies and identify barriers to implementation of public health surveillance case definitions in state NBS programs.
Short-Term Follow-Up	2013–Present	To identify gaps and barriers in short term follow-up education, communication, data collection and reporting in order to inform the development of educational and data sharing initiatives.
Critical Congenital Heart Disease	2013–Present	To identify and share information regarding existing technical assistance and training opportunities for individuals and programs providing Critical Congenital Heart Disease (CCHD) newborn screening
New Disorders	2016–Present	To identify opportunities for technical assistance to be offered specifically regarding the implementation of new disorders that had recently been added to the RUSP.

**Table 2:** NewSTEPs Goals (2014–2018).

Goal 1	Strengthen the newborn screening (NBS) system through enhancement of the existing network of stakeholders by creating a culture of trust, by providing opportunities for timely, interactive communications, and by offering a forum for collaboration among national, regional, and state NBS programs.
Goal 2	Facilitate continuous quality improvement and data-driven outcome assessments in the NBS system by providing a standardized repository and by supporting the integration of health information technology frameworks, including Health Level-7 (HL7) messaging.
Goal 3	Create a dynamic national newborn screening technical assistance resource center that proactively provides training, addresses challenges, and supports program improvement through partnerships with key stakeholders throughout the NBS community.

**Table 3:** Summary Data from 2012 Newborn Screening (NBS) Data Use Survey.

Questions	Response	Response %	Response Count
Do you/does your organization share your NBS data with others outside your agency?	Yes	80%	24
	No	20%	6
Do you/does your organization create standard reports using NBS data?	Yes	75%	24
	No	25%	8
What type of data is included in these reports?	Number of newborns with a specific condition	95.5%	21
	Number of newborn screens completed	100%	22
	timeliness of screens, diagnoses (gap between date of birth and day screened)	54.5%	12
	Number of false positives for screens	68.2%	15
	Other	63.6%	14
Do you have a data system that is working well for you to capture and report NBS data?	No. I/we do not have a data system that works well for us	18.2%	4
	Yes. We use a NBS laboratory information system provided from a vendor to cover all of our data needs	18.2%	4
	Yes. We use a data system developed within our NBS program to meet our data needs	13.6%	3
	Yes. We use a combination system and get data from both the vendor’s system as well as our own data system to collect data	50%	11
	Other	63.6%	14
If you have an existing data system, would you like/be able to transfer data into the NewSTEPs data repository?	Yes, I would like to be able to transfer data to the NewSTEPs data repository	85.7%	18
	No, I don’t want to be able to transfer data to the NewSTEPs data repository, we prefer to enter data for each infant individually	14.3%	3
Are there NBS activities you are doing at the state/regional level that you would like to record/capture in our data system? (e.g., Do you screen for disorders not on the Recommended Uniform Screening Panel (RUSP)? Do you have a call-back program or long-term follow-up program that is not captured in the National Newborn Screening Information System (NNSIS)?)	Yes	50.0%	12
	No	50.0%	12
What NBS technical assistance needs do you have?	Managing newborn screening data	52.4%	11
	Creating reports to share with key stakeholders	66.7%	14
	Communicating with state legislature regarding NBS programs	33.3%	7
	Measuring quality indicators in our state program	52.4%	11
	Trouble shooting laboratory or follow-up challenges in our program	23.8%	5
	Networking with colleagues around the country and in the region to improve NBS outcomes	61.9%	13
	Confirming the diagnosis with subspecialists and clinical providers	28.6%	6
	Other	33.3%	7

**Table 4:** Data Elements Collected Within the NewSTEPs Data Repository.

**State Profiles**	
State Demographics	Number of births (by race, ethnicity, sex)|Number of birthing centers|Number of infants screened|Number of dried blood spot (DBS) specimens received
Disorders Screened	Year disorder was added|DBS collection card image|Testing methodology (1st screen, 2nd screen)|Testing equipment|Target(s) screened|Where testing is performed
NBS Fees	How is screening paid for|Fees for screens|How fees are collected|Services covered by fees
Information Technology (IT) and Laboratory Systems	Applications in use in laboratory and follow-up programs Continuity of Operations Plans (COOP)
Health Information Technology (HIT) Elements	Data integration and exchange policies and procedures
NBS Program Structure	Organizational chart|Hours of operation: laboratory, follow-up|NBS program informational brochures|Contact information: laboratory, follow-up, CCHD, EHDI, HIT|NBS Advisory Committee: Make-up, Charge, By-laws
NBS Policies	Recommended age at initial/second screening, consent, follow-up services, missed cases, birth matching, storage of specimens, storage of data, sharing of specimens, plans during emergencies, addition of new disorders, etc.
**Case Data**	
Demographics	State Unique ID; Date of Birth; Gestational Age; Birth Weight; Biological Sex, Race, Ethnicity
Screening Information	Prenatal testing; Was this individual diagnosed later in life (not identified by newborn screening); Date of specimen collection, receipt by lab, release of out of range results; Date of intervention; Date of confirmation of diagnosis
Case Definition Information	Diagnostic information at the baby level. Data varies by disorder but includes sex, race, gestational age, time elapsed for different NBS services, type of condition.
**Quality Indicator Data**	
Quality Indicators (QIs)	Aggregate data for eight quality indicators

**Table 5:** Newborn Screening Quality Indicators *.

Quality Indicator 1	Percent of dried blood spot specimens that were unacceptable due to improper collection and/or transport.
Quality Indicator 2	Percent of dried blood spot specimens with at least one missing state-defined essential data field upon receipt at the lab.
Quality Indicator 3	Percent of eligible newborns not receiving a newborn screen, reported by dried blood spot or point of care screen(s).
Quality Indicator 4	Percent of infants that have no recorded final resolution (confirmed diagnosis or diagnosis ruled out by an appropriate medical professional) with the newborn screening program.
Quality Indicator 5	Timeliness of Newborn Screening Activities.
Quality Indicator 6	Percent of infants with an out-of-range newborn screen result requiring clinical diagnostic workup by an appropriate medical professional, reported by disorder category.
Quality Indicator 7	Percent of disorders detected by newborn screening with a confirmed diagnosis by an appropriate medical professional.
Quality Indicator 8	Percent of missed cases, reported by disorder.

* Detailed definitions for each quality indicator may be found at www.newsteps.org.

**Table 6:** NewSTEPs National Meetings.

Meeting	Date	Location
Critical Congenital Heart Disease National Meeting	27–28 February 2014	Silver Spring, MD
Collaborative Improvement and Innovation Network for Timeliness National Meeting	15–16 January 2015	Silver Spring, MD
Severe Combined Immunodeficiency National Meeting	30–31 July 2015	Bethesda, MD
Short-Term Follow-Up National Meeting	27–28 October 2016	Orlando, FL
New Disorders National Meeting	22–23 July 2017	Bethesda, MD
Severe Combined Immunodeficiency National Meeting	8–9 August 2017	Washington, DC

## References

[1] Centers for Disease Control and Prevention (CDC) (2012). CDC Grand Rounds: Newborn Screening and Improved Outcomes. Morb. Mortal. Wkly. Rep..

[2] US Department of Health and Human Services Advisory Committee on Heritable Disorders in Newborns and Children: Recommended Uniform Screening Panel.

[3] Association of Public Health Laboratories Newborn Screening Technical assistance and Evaluation Program (NewSTEPs). http://www.newsteps.org.

[4] Government Accountability Office (2003). Newborn Screening Characteristics of State Programs.

[5] Therrell B.L., Panny S.R., Davidson A., Eckman J., Hannon W.H., Henson M.A., Hillard M., Kling S., Levy H.L., Meaney F.J. (1992). U.S. newborn screening system guidelines: Statement of the Council of Regional Networks for Genetic Services (CORN). Screening.

[6] Office of Disease Prevention and Health Promotion (2017). Health People 2020 Maternal, Infant, and Child Health. http://www.healthypeople.gov/2020/topics-objectives/topic/maternal-infant-and-child-health/objectives.

[7] Civic Impulse (2017). H.R. 1281-113th Congress: Newborn Screening Saves Lives Reauthorization Act of 2017. https://www.govtrack.us/congress/bills/113/hr1281.

[8] Therrell B.L., Schwartz M., Southard C., Williams D., Hannon W.H., Mann M.Y., Organizing P.E. (2010). Newborn Screening System Performance Evaluation Assessment Scheme (PEAS). Semin. Perinatol..

